# Supplementary material for: Are carbon emissions trading and green financial instruments synergistic? -Comprehensive quantitative research based on content analysis
Source: PLoS One. 2024 Mar 7;19(3):e0298601. doi: 10.1371/journal.pone.0298601 (PMC10919601; doi:10.1371/journal.pone.0298601)
Supplement: S1 Data — (ZIP) [file pone.0298601.s001.zip › Data/地级市Z_F工作报告.zip!/░▓╗╒/│n╓▌╩╨/└┤╫╘╣1⁄2╓┌║┼í╛┬φ┐╦╩2╛▌═°í┐_2019.docx]

各位代表：

现在，我代表市人民政府，向大会作工作报告，请予审议，并请市政协委员和其他列席人员提出意见。

一、2018年工作回顾

过去的一年，是本届政府的开局之年，也是宏观环境错综复杂、各项工作任务极其繁重的一年。在严峻挑战面前，我们坚持以习近平新时代中国特色社会主义思想为指导，认真贯彻落实党的十九大和十九届二中、三中全会精神，在省委、省政府和市委的坚强领导下，在市人大、市政协的监督和支持下，牢牢把握稳中求进工作总基调，迎难而上、锐意进取，高质量推动五大发展，较好完成了市六届人大一次会议确定的目标任务。

（一）过去一年，是综合实力加快提升的一年。主要经济指标总量稳居全省第一方阵，增速好于预期、快于全省、位于前列。全年地区生产总值增长9.1%；规上工业增加值增长11.4%；固定资产投资增长15.4%；财政收入324.5亿元，增长12.2%；社会消费品零售总额639.1亿元，增长13.5%；外贸进出口31亿美元，增长11.8%；实际利用外商直接投资13.9亿美元，增长13.8%；城镇、农村居民人均可支配收入分别达31230元、13127元，分别增长9.2%、9.9%。总量指标上，地区生产总值、财政收入、外贸进出口居全省前列；动态指标上，地区生产总值、规上工业增加值、固定资产投资、财政收入、社会消费品零售总额、城镇居民人均可支配收入6项指标居全省第一方阵。

（二）过去一年，是改革开放纵深推进的一年。深化改革再创新佳绩。以纪念改革开放40周年为契机，持续推进重点领域改革，共完成120项年度改革任务。农村综合改革走在前列。农村土地“三权分置”改革不断深化，“三变”改革深入推进，获批国家级农村集体产权制度改革试点，在全国会议上作典型发言；小岗喜领全国首批农村集体经济组织登记证，“大包干”带头人被党中央、国务院授予改革先锋称号。全国家庭农场创新发展会议、国有林场改革座谈会、供销总社基层组织建设现场会等在我市召开。综合医改持续深化。“学天长、全覆盖、再创新”深入推进，城乡居民“三保合一”新系统在全省率先上线，市级公立医院改革等顺利完成。“放管服”改革提速提效。滁州和明光通过国家级社会管理和公共服务标准化试点验收。深化“一网一门一次”改革，“一网通办”事项占比99.98%，居全省第一；平均承诺办理时限2.67天，居全省第三；最多跑一次事项占比99.98%。“证照分离”改革试点全面推开，“双随机一公开”监管成效居全省前列。公共资源交易数量、交易金额分别居全省第二、第三。开放合作呈现新局面。13个涉滁事项纳入长三角合作近期工作要点。全市省级以上开发区优化整合为10家，市经开区、苏滁产业园、全椒经开区进入全省30强。全省开发区高质量发展工作推进会来滁观摩。新增进出口实绩企业156家，新批外商投资企业25家，居全省第四。承办了国际隧道与地下空间协会颁奖大会、第十九届万寿论坛等国际性会议。科技创新取得新进展。招才引智“三大行动”深入实施，新签约院士工作站11个，新引进高层次人才团队19个、博士等高层次人才200多人。滁州高新区创建国家级高新区上报待批；与中关村合作的“一中心一基地”、与华为合作的“三朵云一中心”等签约落户。国家级高新技术企业净增数、总数分别居全省第三、第四。产学研合作进一步拓展，累计与78家大院大所建立紧密型合作关系。天长创成全国知识产权强县工程示范县。全柴动力荣获全国质量之光魅力品牌。

（三）过去一年，是实体经济破难奋进的一年。三次产业结构比由14.1:51:34.9调整为12.2:51.7:36.1。工业经济提质提效。新增规上企业103户、总数达1596户，居全省第五，战新产业产值增长19%；亿元以上企业超500户，其中产值超50亿元企业10户，康佳电子产值超150亿元、中普石油超90亿元。通用生物基因合成及测序项目列入省重大新兴产业专项，天康连续8年入选全国电子信息百强，扬子空调等3家企业获批国家级绿色工厂。全力推进西部大工业基地建设，新签约亿元以上工业项目96个，总投资195亿元。明光凹凸棒新材料研究中心成立。现代服务业持续壮大。服务业增加值增长10.5%，居全省第三。旅游业发展步伐加快，全椒慢城、凤阳“四色旅游”建设全面启动，兴茂文旅一期试运营，琅琊山风景区5A级创建上报待审，小岗获评全国百佳乡村旅游目的地。明光包装印刷产业园等3个项目入选省级文化产业示范基地。金融业稳健运行，新增境外上市企业1家，新增本外币贷款347亿元，贷款余额首次突破2000亿元，直接融资146.2亿元。化解非法集资陈案6起。企业债券发行、债券品种创新与风险防范等做法，受到国务院通报表扬。房地产市场保持平稳健康发展，商品房销售面积等6项主要指标居全省前列。建筑业总产值突破430亿元，居全省第五。滁州跨境电商产业园开园运营，华塑物流园获批省级服务业集聚区，广大物流园获批省级示范物流园。现代农业加快发展。粮食生产实现“十五连丰”，全国粮油绿色高质高效现场会、省委农村工作会议等在滁召开。定远国家级农业科技园区通过中期评估，全国村级唯一的小岗国家农业科技园区获批建设，明光农业科技园区入选首批省级农业科技园区。特色农业加快发展，稻虾共作面积实现翻番，突破30万亩，居全省第二；全椒赤镇获批国家级稻渔综合种养示范区；明光绿豆和甜叶菊、全椒龙虾，分别获批国家地理标志保护产品、农产品、证明商标；全年新增“三品一标”产品154个，总数825个，居全省第一。新增家庭农场1300家，总数近8000家，居全省第三。民营经济稳步提升。新增个体工商户39220户、私营企业15727户，省级“专精特新”中小企业达166户，10家、7家、14家企业分别入选安徽民企营收、纳税、进出口百强，天长民营经济考核居全省前列，开润股份入选省十大“单打冠军”。全年共为企业降费2.57亿元，减免税收38.6亿元。“四送一服”双千工程考核连续两年居全省第一。

（四）过去一年，是重大项目取得突破的一年。项目推进工作在全省作典型发言。产业项目上，一批重大项目加快建设。全年新开工亿元以上项目470个。惠科项目加快建设，计划3月份“点亮”；总投资140亿元的康佳智能家电产业园、100亿元的星恒锂电池、50亿元的创维智能家电、50亿元的利维能电池等一批大项目，当年签约当年开工；国能锂电池、隆基乐叶、三棵树涂料等一批项目快速推进。一批重大项目竣工投产。亿元以上工业项目新竣工201个、新达产155个。达亮电子开工8个月即投产，喜星精密开工半年试生产，福莱特一期、嘉安汽车、天鼎丰非织造布、博西华洗碗机、亚士新材料等一批项目竣工投产。一批重大项目签约落户。新签约亿元以上项目446个，总投资220亿元的乌衣古镇、180亿元的金牛湖童世界、3亿美元的华冠通讯等成功落户；阿里巴巴、小米、联想等品牌企业相继进驻。基础设施建设上，滁州人民期盼多年的滁宁城际铁路一期工程开工建设，合新、宁淮高铁在滁境内线形和站点设置基本确定；滁淮高速建成通车，滁天、来六、明巢、滁西环4条高速开工建设，全市高速公路通车里程、在建里程均居全省第一。G104三期建成通车，G328绕城段等开工建设，国省干线建设规模、在建里程和总投资均居全省前列。农村道路畅通工程提前一年完成，绩效考评居全省第一。滁州机场选址有了新进展。淮干蚌浮段治理工程主体完工，女山湖特大桥全线贯通，江巷水库、汊河大桥重建等加快推进。农田水利建设“江淮杯”评比获全省第一。

（五）过去一年，是城乡面貌变化明显的一年。滁城品质持续提升。完成城市空间规划及城市总规修改，划定“三区三线”。大力实施“四个一”工程，琅琊山北门改造、琅琊阁重建等基本完成；清流河水环境综合治理、三期设计等全面完成；古城清流文化街、南谯北路桥和吴棠故居修缮等工程完工，北湖整治初显成效，奠枕楼、拱极门开门迎客，内城河三期、遵阳街及紫薇北路改造等全力推进；明湖建设步伐加快，坝址区园林景观绿化工程全面完成，明湖大道等建成通车，核心区文旅项目、奥体中心等开工建设。镜园、国防训练基地、东坡中学等竣工，儿童医院二期、福利院二期等基本完成，丰收渠综合治理、中都大道北延和城南路网完善工程等全面推进。花鸟鱼虫市场迁建启用。征收房屋295万平方米。文明创建成效显著。扎实开展“十大专项行动”，城市精细化管理有效加强，市民参与度、满意度明显提升，国检省检反映良好。3人荣登中国好人榜，3户获评全国“最美家庭”、“五好家庭”；志愿服务注册率居全省前列。县城和镇村建设步伐加快。县城老城改造、新区拓展和园区提升加快推进，共实施重点项目347个，完成投资150多亿元。天长完成全省美丽乡村整县推进试点，凤阳获批全国美丽乡村建设项目试点。“三大革命”扎实推进，89个乡镇政府驻地建成污水处理设施，改厕35087户，综合整治自然村3604个。全省改善农村人居环境推进会在来安召开。

（六）过去一年，是生态环境持续改善的一年。水清岸绿产业优美丽长江（安徽）经济带、淮河生态经济带建设积极推进，全域推行河（湖）长制、林长制，中央和省环保督察反馈问题年度整改任务全部完成。PM2.5、PM10平均浓度同比分别下降9.8%、1.8%，优良天数同比提升6个百分点。关闭搬迁规模畜禽养殖场389家，完成入河排污口整治243个，7个国控考核断面水质全部达标，县级以上饮用水源地水质合格率100%。第三污水处理厂建成投运。创成省级节水型城市。全椒和南谯分别创成国家级、省级水生态文明城市。启动国家森林城市创建工作，创成省级森林城镇11个、森林村庄60个，完成造林21.3万亩。皇甫山国有林场将军凹入选国家森林小镇建设试点，南谯太平集村入选全国生态文化村。

（七）过去一年，是民生福祉稳步增进的一年。脱贫攻坚迈出新步伐。紧扣“打翻身仗、攻决胜关”目标，以“六大行动”为抓手，深入开展“重补促”专项行动，大力实施“十大工程”，资产收益扶贫“八步工作法”在全省推广，到村到户产业扶贫项目实现全覆盖。新增扶贫小额信贷4.5亿元，居全省前列。定远县摘帽、21个贫困村出列、2.82万贫困人口脱贫有望顺利完成。社会事业实现新发展。城镇新增就业8.9万人、登记失业率2.8%。全省社会救助工作现场会在我市召开。完成农村危房改造13278户，完成率117.4%。学前教育毛入园率93.7%、普惠率79.2%；校外培训机构治理有序推进。市示范性综合实践基地获批全国中小学生研学实践教育营地。中等职业教育质量提升工程中期考核居全省第一。成功举办首届中国农民丰收节滁州分会场活动和第七届中国农民歌会。县级融媒体中心建设实现全覆盖，市图书馆新馆开馆，5个县市图书馆被评为国家一级馆。全椒获批“中国地名文化遗产千年古县”。《滁州市非物质文化遗产保护条例》颁布施行。医疗服务能力进一步提升，建立城市医联体2个、县域医共体20个，获评全国无偿献血先进城市；明光荣获国家基层中医药工作示范单位，琅琊获批省医养结合示范基地。我市在省十四运上金牌数全省第五，首次冲进全省第一方阵；成功申办省十五运和第八届残运会。公共法律服务体系建设实现全覆盖，顺利通过省“七五”普法中期验收。荣膺全省双拥模范城“八连冠”，征兵“五率”居全省前列。十件实事取得新成效。完成棚户区改造10961户；改造老旧小区32个、安置小区20个；升级改造农贸市场11个、迁建3个；完成18个社区公共服务用房达标；改造背街巷道48条；改造公厕73座、新建6座，改造垃圾中转站11座、新建4座；完成主城区水体环境治理5个；新增公办幼儿园3所、普惠性民办幼儿园10所；新建公共停车场2个；完成老年人高龄津贴提标、免费乘坐公交车扩面。社会治理得到新加强。圆满完成村（社区）居民委员会换届选举。在全省率先成立商会人民调解委员会，市县均成立信访事项人民调解委员会。扎实开展“深重促”专项行动和“三转”信访突出问题集中攻坚行动，纵深推进扫黑除恶专项斗争，人民群众安全感居全省第三。安全生产事故起数、死亡人数、受伤人数同比“三下降”。创成全省食品药品安全城市。红十字会、慈善、老龄和残疾人等事业取得新成绩，工会、共青团、妇联、文联、科协等群团组织作用进一步发挥，外事侨务、民族宗教、防震减灾、编制、对台、保密、档案、地方志、工商联、关心下一代等工作全面进步。

（八）过去一年，是自身建设不断加强的一年。坚持把政治建设摆在首位，扎实开展“讲严立”专题警示教育，持续深化“三查三问”，狠抓省委巡视整改。坚持依法行政，深入开展宪法学习宣传教育，严格落实“谁执法谁普法”责任制。推进重大事项合法性审查全覆盖。自觉接受人大法律监督、工作监督和政协民主监督，办理市人大议案3件、建议206件，满意率97.6%；办理市政协提案490件，满意率98.9%；办理市人大常委会审议意见、调研报告等26件，办理市政协资政会建议案、视察报告等16件。坚持正风肃纪，认真履行全面从严治党主体责任，狠抓作风建设，强化审计监督，深入开展政府系统党风廉政建设和反腐败斗争。

各位代表！在经济下行压力加大、困难挑战增多的情况下，取得以上成绩实属不易。这是省委、省政府高度重视、亲切关怀的结果，是市委总揽全局、坚强领导的结果，是市人大、市政协鼎力支持、悉心帮助的结果，也是全市人民奋力拼搏、锐意进取的结果。在此，我代表市人民政府，向全市人民，向人大代表、政协委员和党代会代表，向各民主党派、工商联、无党派人士和各人民团体，向中央及省驻滁单位，向人民解放军、武警官兵、公安政法干警，向所有关心、支持、参与滁州建设与发展的各位老领导、老同志和各位客商、各界朋友，表示衷心的感谢，并致以崇高的敬意!

在肯定成绩的同时，我们也清醒认识到工作中还存在一些问题和不足，主要表现在：发展不足、发展不优、发展不协调仍是现阶段最大的问题，县域经济提升不快、占比不升反降，县际之间发展不平衡；实体经济发展压力较大，部分企业运营成本较高，用工短缺和融资难等问题尚未根本缓解；环境约束趋紧，科技创新和人才支撑不强，新旧动能转换任务依然艰巨；经济社会发展中的风险不容忽视，安全生产、信访维稳等工作还需加强；一些重点领域和关键环节的改革突破不够，基本公共服务还存在一些短板；少数政府部门工作人员担当作为不够，干事创业的状态仍需进一步激发，等等。对此，我们一定高度重视，认真加以解决。

二、2019年工作安排

今年是新中国成立70周年，是全面建成小康社会关键之年。做好今年政府工作，意义重大。

今年工作的指导思想是：以习近平新时代中国特色社会主义思想为指导，深入学习贯彻党的十九大和十九届二中、三中全会精神，落实好中央、省有关会议和市委六届七次全会部署，聚焦“五个坚持”，落实“六稳”要求，全力稳增长、促改革、调结构、惠民生、防风险，扎实开展乡村振兴、创建全国文明城市、县域经济振兴和大江北战略突破年行动，坚定不移冲刺总量全省第三，加快建设现代化新滁州。

经济社会发展主要预期目标是：地区生产总值增长8.5%以上，财政收入增长与经济增长同步，固定资产投资增长13%以上，社会消费品零售总额增长11.5%以上，规上工业增加值增长9%以上，城乡居民人均可支配收入增长明显高于经济增长，城镇新增就业4.2万人，调查失业率控制在5%以内，完成节能减排任务。在实际工作中，要好中加快、能快则快，奋力争先进位（力求主要指标总量和增速进入或靠近全省第三）；各县市区要务求多超（增幅在全市平均水平以上）。重点做好九个方面工作：

（一）聚焦高质量，着力振兴实体经济。

大力发展民营经济。认真贯彻习近平总书记民营企业座谈会重要讲话精神，推动我市民营经济大发展大繁荣。打好政策牌。全面落实省30条、市35条，从市场准入、降低成本、要素保障等方面，尽心尽力为“自己人”办好事，全年要为民营企业减免税费40亿元左右，让民营经济跑出加速度，跨上新台阶。打好扩量牌。全力推动大众创业、万众创新，千方百计做大民营经济“总盘子”。力争新增个体工商户2.2万户、私营企业1万家以上，民营经济占GDP比重66.5%以上。打好升级牌。推进企业产品创新、技术创新、管理创新、模式创新，全力打造一批民营“单打冠军”和“行业小巨人”。力争新增省级“专精特新”中小企业30户以上，争创中国驰名商标2个。打好服务牌。全面开展“进企业门、解企业困、帮企业忙、促企业进”行动，打破各种各样的“卷帘门”、“玻璃门”、“旋转门”，着力构建亲清新型政商关系，让企业创业愉快、兴业愉快、发展愉快。

主攻工业经济。围绕“五大制造”，实施“五大工程”，大力发展先进制造业，推动工业经济实现新突破。实施育大培强工程。强化分类指导，支持金禾实业、开润股份、九华药业等做大做强，力促小企业上规模、中企业上水平、大企业上台阶。全年新增规上企业100户以上、亿元企业20户以上，50亿元和百亿元企业实现再突破。实施改造升级工程。抓好新一轮技术改造三年提升行动，持续推进千亿技改、千企升级，打造一批智能工厂和数字化车间。重点推进5G基站天线、泉盛化工、英发睿能等一批重大技改项目。力争工业投资、技改投资均增长20%以上。实施产业提质工程。以“1+8”省市战新基地为抓手，加快星恒锂电池、国能锂电池、利维能电池、隆基乐叶等重点项目建设，力争战新产业产值突破千亿元。聚焦“互联网+先进制造”，加快创维智能家电、康佳智能工厂、博西华干衣机洗衣机等项目建设，推动智能家电、先进装备等产业迈向中高端。加快非金属矿和农副产品等优势资源高效开发，力促福莱特二期、华塑二期、盼盼小岗食品园等达产达效。实施品牌提升工程。大力推进国家级消费品“三品”战略示范市建设，加快精品培育，打造名企名品。选送一批企业登录央视宣传平台，培育一批省级消费品“三品”示范企业和安徽工业精品。实施绿色增效工程。以节能环保产业“五个一百”专项行动为抓手，加快研发和推广节能环保工艺技术装备，打造一批绿色工厂、绿色产品。

提升现代服务业。实施传统服务业优化提升和现代服务业培育行动，发展新产业、新业态、新模式，力争限上商贸企业突破1000家，服务业增加值增长11%以上。发展文旅康养。加快省级文化产业园建设，推动创源文化、龙利得包装印刷等企业发展壮大，积极争创全省文化企业30强。按照全域旅游思路，依托文旅投融资平台，狠抓“五个一批”工程。积极打造江淮分水岭岭脊生态旅游带，加快推进全椒慢城、来安兴茂、天长金牛湖等一批精品景区和重点项目建设，力争全年完成项目投资90亿元以上。打造大琅琊山文化体验、环滁休闲度假、大明文化和改革源头等一批精品线路。培育亭城文旅、大明旅游、金鹏文旅等一批旅游领军企业。深入实施“旅游+”战略，加快大墅龙山中医药健康旅游示范基地等建设，发展健身康养、科普研学、餐饮民宿等一批旅游新业态。建立旅游产品地方标准体系，促进休闲养生、旅游度假、文化体验等产业融合发展，争创省级医养结合综合示范区。扎实推进“五进”活动，开发一批特色旅游商品，加强旅游市场营销，着力打造区域旅游目的地。力争新创4A级景区2个、琅琊山通过5A级景区创建景观价值国家评定、小岗村完成5A级景区创建景观价值评定国家申报工作，全年游客人次、旅游总收入增长20%以上。做强电子商务。推进京东数字经济产业园、颐高电子商务产业园、创达电子商务城等建设，加快云伙计、网萌、子曰科技等电商企业发展，创建一批省级电子商务示范园区和企业，新增省级电子商务进农村示范县2个。做大综合物流。加快苏滁物流园等建设，积极发展综合物流、专业物流和配送中心，努力构建铁、水、公综合物流体系。做活现代金融。大力实施企业上市培育、产业基金引入、金融生态优化“三大工程”，发展壮大“五大投资平台”，力争新增上市企业2家，新引进股份制银行1家，新增贷款超350亿元，直接融资超100亿元。

加快振兴县域经济。进一步提升县域经济贡献度，力争县域经济总量达1780亿元，占全市比重提高1个百分点以上。强化工业强县，支持各地立足区位、资源等比较优势和产业基础，大力发展特色产业，确保规上工业增加值增长9%以上。强化“双招双引”，确保县域招商引资到位资金超670亿元，新签约亿元以上项目超250个，其中，各县（市）确保引进1个以上超10亿元工业项目；招才引智“三大行动”，各地每项均要有新成果。强化园区建设，大力提升开发区基础设施建设水平和承载能力，鼓励合作共建产业园；促进开发区转型升级，力争再有2—3个园区晋升全省30强。因地制宜，积极推进重点镇加快发展。强化争先进位，进一步完善县域经济考核办法，激励担当作为、比学赶超、竞相发展，力争在全省县域经济考核中，天长保前5，凤阳争前30，其他县（市）前进2位以上。

（二）找准突破口，着力推进乡村振兴。

聚力发展现代农业。坚持“三产”融合，开展“五大行动”。实施农产品加工业提升行动，培育一批农产品加工龙头企业，推进凤阳、明光等4个农产品加工集中区提档升级，力争主营业务收入超120亿元，天长争创全省农产品加工20强。实施特色产业培育行动，重点抓好稻虾共作“双百”工程，今年总面积超50万亩。加快发展“三树一苗”。推进滁菊、芡实、艾草、葛根、百蕊草等中草药产业化，争创国家级滁菊特色农产品优势区、5个省市级特色农产品优势区。实施质量兴农行动，推进高标准农田建设，推广绿色高质高效模式，打造百万亩优质专用品牌粮食生产基地。推进“菜篮子”工程建设。加速建设小岗国家级农业科技园区。实施品牌强农行动，聚焦区域公用品牌和企业品牌“双轮驱动”，新增“三品一标”产品100个以上。各县（市、区）新增1个以上全国全省知名品牌特色农产品。实施功能农业拓展行动，加快培育观光休闲等农业新业态，创建一批田园综合体，开发一批“食药同源”农产品，争创5个以上市级、1个省级现代农业产业园，天长争创国家级现代农业产业园。

大力改善村居环境。学习借鉴浙江“千村示范、万村整治”经验，狠抓“拆、清、治”，一体推进农村环境“三大革命”、“五清一改”清洁家园行动，着力健全长效管护机制，力争完成247个中心村污水处理设施建设，改造危房3800户，改厕31500户，农村生活垃圾无害化处理率达70%以上，村庄整治比例达75%。加快50个省级中心村建设。完成40个乡镇和部分村通天然气。

强力推进乡村治理。健全自治、法治、德治相结合的乡村治理体系，总结推广小岗全国民主法治示范村等经验，开展农村社区建设试点。传承发展乡村优秀传统文化，开展“文明村镇”、“文明家庭”创评，发挥“一约四会”作用，倡导文明健康生活方式，培育文明乡风、良好家风、淳朴民风。

（三）下好先手棋，着力强化创新驱动。

突出平台建设。以“一园三城”为重点，加快国家级产城融合示范区建设，扎实推进高新区创建三年行动计划。苏滁产业园要招引高新技术项目和高层次人才团队5个以上；高教科创城要加快推进中关村发展协同创新中心、长春光机所滁州装备研究院等建设；原创科技城要加快建设京东大数据中心、指南针科创园等；汊河产业新城要依托轨道交通装备产业技术创新联盟，加快产业集聚。推进“三区一城”创建，积极争创国家创新型试点城市。力争新增省级以上研发平台不少于20个，科技孵化器、众创空间5家，各园区科技孵化器、众创空间实现全覆盖。

加快成果转化。加强与高校、科研院所等战略合作，促进更多创新成果落地转化。加快技术转移体系建设，依托中国高校（华东）科技成果转化中心、滁州科技服务联盟等，着力引进一批技术转移服务机构。争取承办省第二届大院大所合作峰会。

强化主体培育。完善“1+4+N”科技创新政策体系，抓好300个技术创新项目、50个研发项目，培育一批创新排头兵。力争新增省级企业技术中心8家、工业设计中心2家、高新技术企业50家，年开发新产品200个以上。

重抓人才集聚。落实人力资源改革“1+3”政策。多管齐下引才，力争新签约院士工作站10个，新引进高层次科技人才团队15个、博士等高层次人才200人。固本培元育才，深化校地合作，积极构建由企业、高校、科研院所共建共享的人才培养平台，加大高技能人才培养力度，分批选送优秀企业负责人到长江商学院等院校学习。想方设法留才，落实好引进人才及聘用务工人员住房等优惠政策，着力解决好人才落户涉及的住房、医疗、子女入学等难题，完善人才服务保障体系。各尽其能用才，用好用活“编制周转池”，分类推进人才评价机制改革，最大限度激发和释放人才创新创业活力，让创新源泉充分涌流。

（四）牵住牛鼻子，着力抓好项目工作。

全力招项目。坚持高位推动，强化以商招商、专业招商、园区招商，着力引进一批龙头型、科技型、高成长型项目。力促芯片晶圆、电子基板玻璃、新能源汽车等一批重大项目签约落地，确保引进市外亿元以上项目超400个，到位资金突破1000亿元，力争在百亿级项目引进上再有新突破。

全速建项目。进一步提高项目开工率、竣工率、投产率和达产率。产业项目上，开工建设日泰汽车标准件、华冠通讯等亿元以上项目300个；加快推进胜华波微特电机、金轩科技等320个在建项目；力促亚玛顿、长荣光纤光缆等200个项目竣工投产。基础设施项目上，推进滁宁城际铁路一期加快建设、二期开工。加快滁天、来六、明巢、滁西环4条高速建设，确保合宁高速四改八建成通车。力促合新、合宁、宁淮高铁和宁洛高速改扩建项目开工建设。实施516公里国省干线建设工程、3271公里农村公路扩面延伸工程，扎实推进“四好农村路”建设。加快滁州机场和中海油天然气热电厂工程前期工作。建成江巷水库，争取滁河新增治理工程开工。500千伏同乐变、220千伏会峰变等建成投运。

全面谋项目。抢抓国家战略、宏观政策、开放发展等机遇，聚焦重大平台、重大基础设施、重大投资专项等，精心谋划高端制造、科技创新、基础设施、民生改善等一批项目。争取亿元以上项目储备超1000个，更多项目挤进国家和省盘子。

全程帮项目。巩固深化“四送一服”成果，市县两级建立企业帮扶中心，全力创优“四最”营商环境。对重大项目实行全过程管理、一体化跟踪、保姆式服务，做到领导牵头帮扶项目、平台调度项目、督查机构督查项目、各方合力推进项目，着力为项目建设保驾护航。

（五）突出主抓手，着力建设美丽家园。

突出空间管控，提升规划水平。对接长三角城市群发展规划，以国土空间规划为统领，抢抓合肥、南京都市圈新一轮规划修编机遇，优化市域空间布局，高标准编制滁来全城镇群道路交通、市政设施、生态绿地等专项规划。结合城乡规划体制改革，完善“多规合一”一张图和信息化平台，全力推进城市设计和特色风貌打造。完成县域乡村建设规划及特色小城镇规划编制。全市城镇化水平提升到56%。

突出功能配套，提升建设水平。滁城建设上，聚焦“四个一”工程，实施重点城建项目260个以上，完成投资200亿元。琅琊山要依托环琅琊山风景道、环西涧湖景观道，实施好清丰路、琅琊山南门等项目，彰显山水风光；清流河要加快紫薇路桥、清流河三期四期等工程建设，做美水景观；古城北湖综合整治和东大街桥竣工，遵阳街改造主体完工，太仆寺和吴棠、章益故居完成布展，积极创建国家级历史文化名城；明湖片区要着力推进文旅PPP项目，实施胜天河上下游水系治理、“四横三纵”路网、龙兴路及绿廊等重点项目，加快奥体中心、明湖中学等重大项目建设。县城建设上，推进产城融合、功能配套、管理提升，加快构建特色鲜明、设施完善、和谐宜居的新型城镇。特色小镇建设上，结合乡村振兴，建设一批特色小镇，着力打造汊河国家级特色小镇，冶山玩具等省级特色小镇，明光凹土等8个市级特色小镇。

突出文明创建，提升管理水平。大力推进全民创建，确保年度测评居全省前列。加快大改造。持续推进老旧小区、棚户区、农贸市场、公厕、城乡结合部等改造，力争滁城房屋征收面积超200万平方米。实施大整治。深入推进交通秩序、经营秩序、文明养犬、餐饮油烟、河湖水体、村居环境“六大专项整治”行动，进一步巩固环卫保洁一体化和“一街四方”联勤共治成果。促进大提升。统筹推进中国人居环境奖、国家节水型城市等多城联创，加快创建新型智慧城市示范市。深入推进社区“六个一”建设，完善网格化管理机制，探索“楼长制”、“街巷长制”，深化文明单位结对创建，进一步提高群众参与度，合力提升城市管理水平。

（六）打造新引擎，着力建设大江北协同发展区。

区域规划上对接衔接。围绕当好江北新区的拓展腹地、生态屏障、产业配角，加强“一主四副”及天长空间规划整合，加快编制毗邻江北新区扇形区域相关规划，实现近期建设区域控制性详细规划及城市设计全覆盖，积极构建大江北地区“1+3+5+N”空间发展格局。

基础设施上互联互通。围绕构建与江北新区半小时通勤圈，加快以高铁、城际、高速、国省干线和快速通道为骨干的综合交通网互联互通。推进东部干线、滁宁快速第二通道、连接南京北站等快速通道的规划建设，加快实施襄河航道整治、汊河集船闸等水运工程，打造综合交通体系，推进公共交通一体化。

产业发展上协同协作。对接江北新区“两城一中心”，谋划打造宁滁合作园区，规划建设一批中小企业园和产业集中区。加大配套产业项目招引和承接力度，加强与江北新区产业链式合作，推进产业错位发展、协同发展，培育我市新的经济增长极。

公共服务上共建共享。积极推进与南京合作办学、联合办医，加强医保定点、异地就医即时结算等方面合作。继续推进社会保险转移平台建设，大力推广“互联网+社保”，加快推进智能卡在交通、社保、旅游等更多领域共享共用。

（七）激活动力源，着力深化改革开放。

改革再出发。坚持抓小岗促全市，大力推进重点领域和关键环节改革。机构改革上，全面做好人员转隶、机构挂牌、机关“三定”等工作，确保3月底前完成市县机构改革任务。农村综合改革上，争创国家农村改革试验区。全面完成农村集体产权制度改革国家级整市试点；加快农村“三变”改革，发展壮大村级集体经济。探索宅基地“三权分置”，推进农村土地股份合作。综合医改上，健全完善全民医保、药品供应、分级诊疗、现代医院管理等制度，完善医疗卫生服务体系，进一步破解“看病难、治病贵、防病弱”等问题，打造滁州医改升级版。“放管服”改革上，推广“网上办、集中批、联合审、区域评、代办制、不见面”审批模式，深化“一网一门一次”改革，让群众和企业办事从“面对面”到“键对键”。新型城镇化改革上，加快人力资源、住房、教育、社保等方面政策协同，推动已经在城镇就业农业转移人口落户，吸引外出务工人员、大中专毕业生、中西部市县富余劳动力“三支力量”来滁就业创业，增强城镇特别是滁城的人口集聚力。同时，统筹推进生态文明、社会事业等领域改革，不断增添发展新动力。

开放再扩大。抢抓长三角更高质量一体化发展机遇，强化“双圈”互动，做好融通大文章。积极融入“一带一路”，借助国家发改委国际合作中心等平台优势，面向全球开展产业链招商，着力引进一批世界知名企业；培育壮大外贸主体，做实滁州跨境电商产业园，精心组织参加第二届中国国际进口博览会，确保新增进出口实绩企业100家以上；大力支持企业“走出去”，确保实际利用外商直接投资增长7%以上，外贸进出口增幅高于全国平均水平。

园区再发力。市经开区要自我加压，力争新签约、新开工、新竣工亿元以上项目分别超50个、28个、30个，园区总营收超1500亿元，亿元以上企业超60户，50亿元以上企业超6户。苏滁产业园要聚焦“攻坚百个项目、实现百亿投入”目标，力争新签约、新开工、新竣工亿元以上项目分别超40个、35个、30个，亿元以上企业超12户。市经开区、苏滁产业园还要力争50亿元以上工业大项目再有新突破。西部大工业基地要进一步增强园区的承载力和牵引力。其他开发区要加快园区扩容升级、提质增效。

（八）突出补短板，着力打好三大攻坚战。

打好防范化解重大风险攻坚战。建立健全金融监管体系，深入开展非法集资、互联网金融、交易场所等专项整治行动，规范小贷公司、融资担保公司等发展，加强政府债务管理，有效防范化解政府债务风险，坚决守住不发生系统性金融风险的底线。

打好精准脱贫攻坚战。认真开展中央专项巡视交办问题集中整改，确保“严规矩、强监督、转作风”行动取得实效。深入推进“十大工程”，深化“四带一自”产业扶贫、“三有一网”点位扶贫、“三业一岗”就业扶贫，打造产业扶贫、就业扶贫升级版。完善“351”“180”健康脱贫政策。深化结对帮扶和定点扶贫，确保再脱贫1.3万贫困人口。构建防范返贫、稳定脱贫有效机制，将返贫户和新增贫困户及时纳入帮扶范围，做到既实现高质量脱贫、又织密防范返贫保障网。

打好污染防治攻坚战。坚持铁腕治污。全面做好大气、水、土壤污染防治工作，持续推进环保督察反馈问题整改。开展机动车尾气、工业锅炉等专项治理，抓好滁城烟花爆竹禁放，建成大气网格化监控系统，持续改善空气环境质量，确保完成省下达的PM2.5年度目标任务。推深做实河（湖）长制，开展滁河跨省水质生态补偿试点，严格执行市域地表水生态补偿制度。强化国控断面和入河排污口水质监测，消除18个城市黑臭水体，大力推进海绵城市建设。加强农业面源污染防治，确保化肥农药“零增长”、秸秆综合利用率超90%、畜禽粪污综合利用率超75%。加强固体废弃物污染防治和污染地块风险管理，实施土壤治理与修复试点项目。筑牢生态屏障。以创建国家森林城市为抓手，加快林长制“七个一批”建设，推进“十百千万”国土绿化、绿色滁州等四大行动，完成营造林83万亩，力争创成省级森林城市、生态县各1个，森林覆盖率达35%以上。严守生态红线。牢牢守住生态保护红线、永久基本农田、城镇开发边界三条控制线，强化“大棚房”和违规殡葬设施整治，严格实施能源消耗、水资源消耗、建设用地总量和强度“双控”制度。

（九）织密民生网，着力提升幸福指数。

优先发展教育事业。扎实推进第三期学前教育行动计划，新建、改扩建公办幼儿园19所。大力实施义务教育质量提升行动，深入推进新一轮义务教育学校标准化建设，新建醉翁路小学等中小学11所。落实全国中小学生减负30条措施。支持安徽科技学院、滁州学院加快建设地方应用型高水平大学；支持职业技术学院、城市职业学院加快建设地方技能型高水平大学。加快推进滁州机电工程学校等11所中职学校建设。争创省级产教融合示范试点城市。

加快打造健康滁州。深化拓展县域医共体和家庭医生签约服务，抓好中心卫生院管理圈和中心药房试点，把更多救命救急的好药纳入医保。推进“互联网+医疗健康”，建设全民健康信息平台，加强社区卫生服务中心建设，抓好天长、凤阳“智医助理”试点。完善全面两孩政策配套。加强全科医生队伍建设。力争创成省级卫生城市。

繁荣发展文化事业。深入推进文化惠民工程，建好“四个十”文化基础设施，推进第四批全国公共文化服务体系示范项目创建。开展戏曲进校园、全民阅读、送电影下乡等活动。启动修缮柏子龙潭。力争琅琊山无梁殿等获批国家级文保单位。

完善社会保障体系。重点抓好高校毕业生、农民工、退役军人等群体就业，动态消除零就业家庭。推进农民工工资“治欠保支”。深入实施全民参保计划。推动城乡居家养老三级中心建设，探索智慧社区养老示范中心、医养结合等试点。持续推进住房公积金建制扩面。抓好省级乡镇政府服务能力建设试点。

维护社会和谐稳定。继续推进扫黑除恶专项斗争，持续开展打击犯罪、维护稳定、守护平安等系列专项行动。落实信访工作责任制，深入开展“三转”突出信访问题集中攻坚行动。全面落实食品药品、农产品安全源头治理，全力保障群众饮食用药安全。严密防控重大动物疫情。严格落实《党政领导干部安全生产责任制实施细则》，深入推进安全生产“铸安”行动常态化实效化和风险管控“六项机制”制度化规范化，认真落实“路长制”，全力遏制重特大事故。加快“雪亮工程”建设，推进市县乡村四级综治中心标准化建设，织密社会治安防控网络，进一步深化平安滁州建设。

统筹做好各项工作。以全民健身为重点，推进体育强市建设，力争实现县、乡公共体育设施“五个一”、“三个一”全面达标。积极筹备省十五运、第八届残运会、第五届全民健身运动会，精心承办奥运会女子手球亚洲选拔赛。加强国防动员和后备力量建设，推动军民融合深度发展，做好退役军人服务管理，争创全国双拥模范城“六连冠”。推进残疾人康复中心和残疾人之家建设。抓好第四次全国经济普查，做实数据、摸清家底。支持工会、共青团、妇联、文联、侨联、科协等群团工作，进一步加强外事、民族宗教、保密、人防、地震、气象、老干部、地方志、红十字会等工作。

努力办好十件实事。今年市委、市政府在滁城继续为市民办好十件实事：改造棚户区6500户；整治改造老旧小区26个；改造背街小巷59条；新建改造公厕43座；新建改造农贸市场14个；实施23个社区公共服务用房达标工程；新建公共停车场3个、临时停车场10个；建设改造公共体育公园12个、为26个安置小区增配全民健身器材；实施餐厨和厨余垃圾处理项目；重建、改造危桥4座。

三、加强政府自身建设

自觉以习近平新时代中国特色社会主义思想武装头脑、指导实践、推动工作，着力加强政府自身建设，全面提高履职水平。

不忘初心，牢记使命。坚持把党的政治建设摆在首位，始终保持斗争精神，扎实开展“不忘初心、牢记使命”主题教育，树牢“四个意识”，坚定“四个自信”，坚决做到“两个维护”。坚持以人民为中心，始终把人民对美好生活的向往作为我们的奋斗目标，践行党的根本宗旨，贯彻党的群众路线，让人民共享发展成果，有更多、更直接、更实在的获得感、幸福感、安全感。

依法行政，严以用权。增强宪法意识，自觉做社会主义法治的忠实崇尚者、自觉遵守者、坚定捍卫者。认真贯彻民主集中制，加强政府重大事项合法性审查规范化建设，提高政府决策科学化、民主化、法治化水平。认真落实市人大及其常委会决议决定，自觉接受人大法律监督、工作监督和政协民主监督，主动听取民主党派、人民团体和社会各界意见建议，提高人大议案、代表建议和政协提案办理质量。严格执行向市人大常委会报告国有资产管理情况等制度。深化政务公开，加强信息发布和政策解读，及时准确回应社会关切，让权力在阳光下运行。

勇于担当，实干兴滁。崇尚实干、力戒空谈，努力奔跑、奋力追梦，切实把心思集中在“想干事”上，把担当体现在“敢干事”上，把能力展示在“会干事”上，把业绩记录在“干成事”上，以坚如磐石的信心、只争朝夕的劲头、坚韧不拔的毅力、求真务实的作风，踏石留印、抓铁有痕，让改革发展稳定各项任务落下去，让惠及百姓的各项工作实起来。

率先垂范，廉洁从政。认真履行全面从严治党主体责任和“一岗双责”，强化巡视巡察、环保督察和审计结果运用，严肃查处侵害群众利益等突出问题，坚决惩治各类腐败行为。认真落实中央八项规定精神和省市相关要求，严格执行领导干部廉洁从政各项规定，从严整治“四风”问题特别是形式主义、官僚主义，坚决打好作风建设持久战。明大德、守公德、严私德，努力实现干部清正、政府清廉、政治清明。

各位代表、同志们，实干成就梦想，奋斗铸就辉煌。让我们更加紧密地团结在以习近平同志为核心的党中央周围，在省委、省政府和市委的坚强领导下，戮力同心，砥砺奋进，为冲刺总量全省第三、决胜全面建成小康社会、加快建设现代化新滁州而努力奋斗！
